# Acupuncture for dyspnea and breathing physiology in chronic respiratory diseases: A protocol of a systematic review and meta-analysis of randomized controlled trials

**DOI:** 10.1097/MD.0000000000030909

**Published:** 2022-10-14

**Authors:** Chan Xiong, Yu Li, Chen-Yi Li, Ye-Fang Liu, Hua Wei, Juan-Juan Fu

**Affiliations:** a Department of Respiratory, No. 3 Affiliated Hospital of Chengdu University of TCM (West District), Chengdu Pidu District Hospital of TCM, Chengdu, Sichuan, China; b Department of Research, No. 3 Affiliated Hospital of Chengdu University of TCM (West District), Chengdu Pidu District Hospital of TCM, Chengdu, Sichuan, China; c Department of Integrated Traditional and Western Medicine, China Hospital, Sichuan University, Chengdu, Sichuan, China.

**Keywords:** acupuncture, chronic respiratory diseases, dyspnea, protocol, systematic review

## Abstract

Dyspnea is one of the most common symptoms of chronic respiratory disease (CRD) and is closely related to increased functional disability and mortality, resulting in substantial adverse outcomes on patients and imposing great social and economic burden. Although multiple clinical trials and systematic reviews have suggested that acupuncture could be effective in treating COPD and lung cancer, little is known about its effects on dyspnea relief in patients with CRD. The present study aimed to use a systematic review approach to evaluate the effectiveness and safety of acupuncture in the treatment of dyspnea in patients with CRD.

**Methods:**

We will search the following 9 databases from inception to June 30, 2022, PubMed, Web of Science, EMBASE, Cochrane Central Register of Controlled Trials, Chinese National Knowledge Infrastructure, WANFANG Database, Chinses Scientific and Technological Periodical Database, and Chinese Biomedical Database, and the Cochrane Library Database. Clinical randomized controlled trials in English or Chinese that evaluate invasive acupuncture versus control group in treatment of CRD with dyspnea will be included. The primary outcome will be dyspnea scores, breathing physiological function, and the secondary outcomes include exercise tolerance by six-minute walk distance quality of life, quality of life and adverse events. Two reviewers will independently conduct study selection, data extraction and quality assessment. The Review Manager software will be used for meta-analysis. This protocol will be carried out in accordance with the PRISMA-P guidance.

**Conclusion:**

This systematic review and meta-analysis will provide the evidence of whether acupuncture is an effective and safe intervention for CRD with dyspnea. The results will be disseminated through peer-reviewed publication.

## 1. Introduction

Chronic respiratory diseases (CRD), describing a range of disease of the airways and the other structures of the lungs in which chronic obstructive pulmonary disease (COPD), asthma, interstitial lung disease, lung cancer, bronchiectasis, cystic fibrosis, sleep apnea, occupational lung diseases and pulmonary hypertension are the most common. It has been estimated that close to 545 million people worldwide had a chronic respiratory disease in 2017, an increase of 39.8% since 1990. CRD has imposed an immense health burden as the third leading cause of mortality, accounted for 3.9 million deaths per year, and was also associated with disability and enormous economic burden.^[1]^ Effective treatments to control symptoms, improve patients’ quality of life and prevent adverse outcomes will warrantee reduction in morbidity, disability and risk of death.

Dyspnea is one of the most common and prominent symptoms suffered by patients with CRD leading to varying intensity of discomfort and distress. More importantly, dyspnea is potent predictor of mortality,^[2]^ often surpassing common physiological measurements in predicting the clinical course of a patient^[3]^ such as decreased exercise tolerance and health-related quality of life, and increased risk of hospitalization.^[4–7]^ Treatment of the underlying cause of dyspnea is the most direct approach to ameliorating the symptom, but there are many patients for whom dyspnea persists despite optimal treatment.^[8]^

Although our understanding of the pathophysiology of dyspnea has been significantly improved, it only has been modestly translated into the advances in the therapies to symptomatic patients. Research on pharmacological and nonpharmacological approaches, for example, anxiolytics, antidepressants, oxygen, pulmonary rehabilitation, yielded ineffective or conflicting results, or focused on specific disease with limited indication.^[9]^ Opioids administration reduce dyspnea in advanced COPD, interstitial lung disease, cancer, however, it is associated with frequent side effects.^[10]^

Discovery of other therapeutic strategy to ameliorate the frequently occurred dyspnea in a wide spectrum of CRD is in pressing need. Traditional Chinese medicine, as an alternative and complementary therapy for western medicine, plays an important role in preventing and treating various chronic diseases, in which acupuncture is a widely used non-pharmacological therapy for CRD. A series of randomized controlled trials (RCTs) have evaluated the effectiveness of acupuncture on relieving dyspnea in COPD.^[11–13]^ A milestone for the recognition of applying acupuncture in CRD is that the 2021 update of global initiative for chronic obstructive lung disease document firstly acknowledged acupuncture as an approach in patients with advanced COPD which may improve breathlessness and quality of life.^[14]^ Acupuncture improved breathlessness severity in patients with advanced diseases including lung cancer in a recent study,^[15]^ however, this study included breast cancer while studies reported in Chinese were not addressed. Improvement of symptom was also observed for acupuncture in asthma.^[16]^ Therefore, acupuncture has shown promising treatment benefits in a variety of CRD. Although dyspnea in different CRD is associated complex and different pathophysiological abnormalities, common pathogenesis of dyspnea exists among these diseases^[17]^ such as neuromechanical dissociation^[18]^ and affective distress by which anxiety, panic and depression can affect the experience of dyspnea,^[19]^ where the treatment effects of acupuncture may underlie.

However, due to the uneven quality of studies and selection of different clinical outcomes, there are insufficient evidence and a series of uncertainty about the effects of acupuncture in the treatment of CRD. Whether it can improve dyspnea and breathing-related objective parameters such as lung function and exercise tolerance in CRD are unsolved but imperative questions which hampered the application of acupuncture in CRD. There is no published systematic review and meta-analysis to address the above questions so far. In this study, we intend to access a comprehensive category of CRD and systematically evaluate the efficacy of acupuncture in the treatment of CRD on dyspnea and breathing function.

## 2. Methods

### 2.1 Study registration

This protocol has been registered on international prospective register of systematic review (PROSPERO) as CRD42020189624 (Available from: https://www.crd.york.ac.uk/prospero/#recordDetails). It will be conducted following the guideline of Preferred Reporting Items for Systematic Reviews and Meta-analyses Protocols (PRISMA-P).

### 2.2 Criteria for including studies in this review

#### 2.2.1 Types of studies

RCTs that evaluated any type of invasive acupuncture will be included, irrespective of blinding. Acupuncture therapy including body acupuncture, warm needing and electroacupuncture.

#### 2.2.2 Participants

1. Adult participants (≥18 yr of age) with dyspnea due to CRD, regardless of their gender, race, education or economic status.
2. The types of CRD were as following (any stage, if associated with dyspnea): COPD, emphysema, lung cancer, asthma, bronchiectasis, interstitial lung disease, chronic pulmonary heart disease, and bronchitis.

#### 2.2.3 Exclusion criteria

1. Randomized crossover trials, reviews, case-control, case series, qualitative studies, case reports, or animal experiments;
2. Combined with other intervention of traditional Chinese medicine, such as oral Chinese herb medicine, cupping, catgut embedding at acupoints, etc.;
3. Studies investigating other methods of stimulating acupuncture points without needle insertion (e.g., acupressure, pressed studs, laser stimulation, cupping or transcutaneous electrical stimulation) will be excluded;
4. Patients with significant diseases other than CRD mentioned above, including a diagnosis of congestive heart failure, as well as severe complications and complications of other organs.

#### 2.2.4 Interventions and comparators

The intervention measures of the treatment group will be administrated with acupuncture based on routine treatment of western medicine. The intervention measures of the control group will be treated with routine western medicine or sham acupuncture combined with routine western medicine.

#### 2.2.5 Outcome

Primary outcomes:

1. Severity of Dyspnea by scores including the visual analogue scale,^[20]^ the Borg scale score, the modified Medical Research Council scale,^[21]^ and the COPD assessment test;^[22]^
2. Breathing physiology:^[23]^ change in lung function tests including forced expiratory volume in one second, forced vital capacity, and peak expiratory flow.

Secondary outcomes:

1. Exercise tolerance: 6-minute walk distance;^[24]^
2. Quality of life: measured by a validated questionnaire, for example, St. George’s Respiratory Questionnaire;^[25]^
3. Adverse events.

### 2.3 Search strategy

The search languages are English and Chinese, and the following electronic databases will be searched from their respective inception to June 31, 2022: PubMed, Web of Science, EMBASE, Cochrane Central Register of Controlled Trials (CENTRAL), Chinese National Knowledge Infrastructure (CNKI), WANFANG Database, Chinses Scientific and Technological Periodical Database (VIP), and Chinese Biomedical Database (CBM), and the Cochrane Library Database. Additionally, we will search databases of ongoing registered clinical trials including Chinese clinical trial registry (http://www.chictr.org.cn/) and international clinical trial registry (http://clinicaltrials.gov/). Moreover, we will also manually search the additional relevant studies, using references of the previously published systematic reviews. Search terms will be used alone or in varying combinations. Table 1 provides the search strategies in the PubMed database, and other databases will use these strategies similarly. The Chinese database will also use search terms with the equivalent English meaning.

**Table 1:** Search strategy used in PubMed database.

Number	Search items
1	Randomized controlled trial (RCT)
2	Controlled clinical trial
3	Randomized
4	Randomly
5	Trial
6	1 or 2–5
7	Chronic obstructive pulmonary disease (COPD) or
8	Emphysema
9	Lung cancer
10	Asthma
11	Bronchiectasis
12	Chronic pulmonary heart disease
13	Bronchitis
14	Interstitial lung disease (ILD)
15	7 or 8–14
16	Acupuncture
17	Acupoint
18	Meridian
19	Electroacupuncture
20	Fire needle
21	Warm acupuncture
22	16 or 17–21
23	6, 15 and 22

### 2.4 Study selection and data extraction

**Figure 1.**
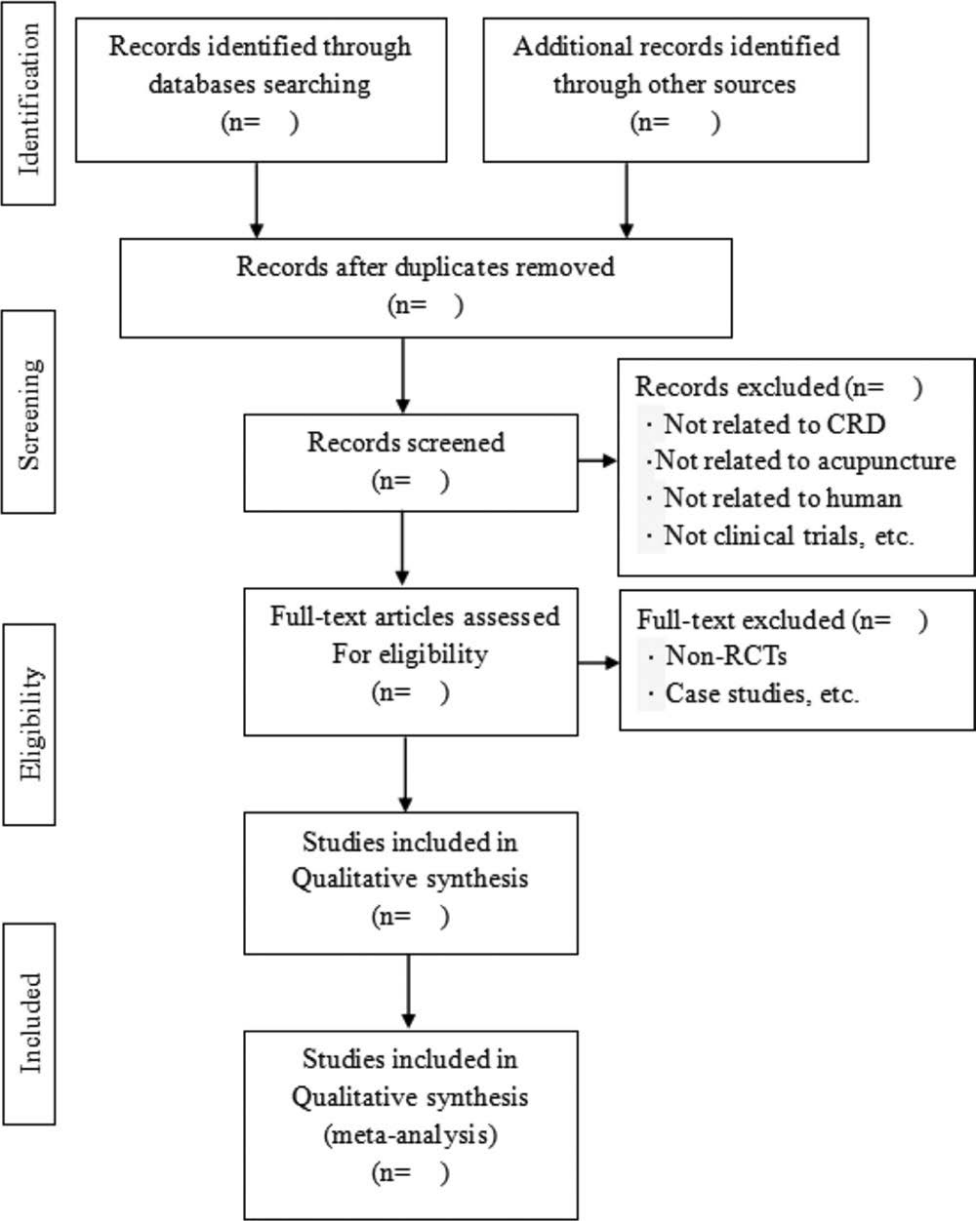
Flow diagram of the trial selection process. CRD = Chronic respiratory diseases, RCTs = randomized controlled trials.

Each title and abstract will be evaluated by two independent reviewers according to the predefined inclusion and exclusion criteria. The further screening will be performed to select eligible articles by reviewing the full text. All rejected articles will be confirmed by another reviewer. For each excluded study, a specific reason for exclusion will be provided and validated by a third reviewer. If necessary, a third reviewer will be consulted to resolve any differences. The selection process will be summarized in a PRISMA flow chart (Fig. 1).

We will design standardized database tables for data extraction. Data extraction items include: title, first author, publication year, country, diagnose information, disease duration, stage, sample size, age, details of intervention, control and outcomes, treatment duration and follow-up period and adverse events. Data will be extracted independently by two reviewers, and all extracted data will be cross-checked by two reviewers to ensure accuracy. Differences in the extraction process will be resolved by a third reviewers.

### 2.5 Risk of bias assessment

The methodological quality of included studies will be assessed using the tool for “risk of bias” from the Cochrane Handbook for Systematic Reviews of Interventions.^[26]^

The assessment details as following:

1. Random sequence generation.
2. Allocation concealment.
3. The use of blindness.
4. Incomplete outcome data.
5. Selective reporting.
6. Other sources of bia.

Each item will be assessed as “high risk”, “low risk”, or “unclear risk” according to the details of eligible studies. For any unclear items in the study, contact the corresponding author for details. Any disagreement will be resolved by discussion with a third reviewer.

## 2.6 Statistical analysis

For each outcome, we will use Review Manager 5.3 provided by Cochrane collaboration for meta-analysis. Dichotomous data will be analyzed using the relative risk ratio (RR) of 95% confidence interval (CI) and *P* value. Continuous data will be analyzed using the mean difference (MD) of 95%CI and *P* value. *P* < .05 will be considered statistical significance. Statistical heterogeneity will be analyzed by χ^2^ test, and I^2^ value will be used to estimate the size of heterogeneity. When the heterogeneity test shows that there is heterogeneity among the multiple included studies (*P* ≤ .05, I^2^ ≥ 50%), the heterogeneity source can be confirmed firstly through subgroup analysis or sensitivity analysis, and if heterogeneity source cannot be judged or eliminated, the random effect model is used for combined analysis. When the heterogeneity test shows that there is no heterogeneity among the multiple studies included (*P* > .05, I^2^ <50%), the fixed effect model will be used for combined analysis. If quantitative synthesis is not appropriate, qualitative analysis will be carried out. Sensitivity analysis will be conducted to evaluate the robustness of the meta-analysis results.

### 2.6.1 Dealing with missing data protocol

If missing data are detected, we will contact the author to obtain the necessary information. If not obtained, we will use the existing data for analysis and discuss the possible impact of missing data on the results.

### 2.6.2 Publication bias

We will use funnel plot to detect potential publication bias, if more than 10 trials are included in the study. The Egger regression test will be used to determine the asymmetry of the funnel plot.^[27]^

### 2.6.3 Subgroup analysis

If significant heterogeneity is detected between studies, a subgroup analysis will be performed. Factors such as the type control such as sham control, type of stimulation (manual vs electric), duration of treatment, treatment frequency, measurements of results, severity of disease, age, sex, geographic area will be considered.

### 2.6.4 Quality of evidence

The GRADE (the Grading of Recommendations Assessment, Development and Evaluation) approach will be used to assess the evidence quality of the primary outcomes, which will be divided into four levels: very low, low, medium, or high.^[28]^

## 3. Discussion

The prevalence of mild to moderate dyspnea was 9-13% among adults living in the community, and 15% to 18% among those adults aged 40 years or older, and 25% to 37% of adults aged 70 years and older.^[2]^ The proportions are even higher in patients with CRD, in which 53% of patients with COPD still present severe breathlessness despite optimal inhaled medications^[29]^ and up to 74% of patients with lung cancer experience dyspnea,^[30]^ with major impact on the quality of life of the patients, their family and the caregivers.

It is not only a clinical symptom complained by the patients, importantly, it is an important predictor of mortality, often surpassing common physiological measurements in predicting the adverse clinical outcomes of a patient,^[18]^ such that dyspnea is a better predictor of 5-year survival than airway obstruction in patients with COPD.^[31]^ A study involving 3646 patients aged 65 and over found that dyspnea was closely related to the increase of long-term mortality in the elderly population regardless of BMI and other mortality factors.^[32]^ Dyspnea also imposes the suffered patients with an adoption of sedentary lifestyle which predictably leads to extensive skeletal muscle deconditioning, social isolation, negative psychological sequalae. Social isolation or withdrawal may breed discouragement, frustration, loneliness, or depression, which may further decrease functional performance and quality of life.^[18]^

In clinical practice, medications commonly used for dyspnea include opioids, benzodiazepines, antidepressants, etc.^[2]^ However, the use of these drugs has obvious limitations. For example, opioids can be used to treat refractory dyspnea in COPD, interstitial lung disease and advanced lung cancer, but it is necessary to pay attention to its side effects such as nausea, vomiting and constipation.^[33]^ Benzodiazepines can cause drowsiness and somnolence,^[34]^ while a Cochrane systematic review found no convincing evidence for or against the use of benzodiazepines for chronic dyspnea.^[34]^ Exploration on alternative therapy with proved clinical efficacy and less side effects is warranted.

Acupuncture as an essential component of alternative and complementary medicine has been accepted by the mainstream of contemporary medicine worldwide given its wide indications, reliable curative effect, convenient operation, economical and safety. It is widely practiced for a wide spectrum of diseases ranging from chronic pain, migraine high blood pressure to cancer beyond its theoretical basis of balancing the flow of energy or life force, known as qi (chi), by diverse needling techniques to activate the meridians and acupoints of the body. Treatment effects of dyspnea with acupuncture begin to be realized based on recent study findings, while this is a new area for acupuncture that needs much more robust evidence and elucidation of underlying mechanisms, like chronic pain, which will popularize the application of acupuncture.

Although specific mechanisms for acupuncture in treating CRD with dyspnea is not well established, a series of findings provide theoretical and mechanistic basis for the treatment effects of acupuncture in relieving dyspnea. The levels of β-endorphin, an endogenous opioid peptide that modulate dyspnea and pain, were increased after transcutaneous electrical nerve stimulation over acupuncture points in patients with COPD.^[35]^ Acupuncture may also play a neuro-endocrine regulatory role by activating vagus nerve and decreasing the release of acetylcholine in lung, acting as bronchidilating and anti-inflammatory activities.^[36]^ In addition, acupuncture may suppress inflammatory responses by reducing levels of inflammatory mediators such as IL-8, IL-1β,IL-6 and tumor necrosis factor-α.^[37,38]^ The negative affective distress in patients complained dyspnea aggravates the severity of dyspnea, while acupuncture may exert its therapeutic effect by processing on the activated cerebral regions for both pain and dyspnea.^[39]^

The clinical efficacy of acupuncture for dyspnea has not been assessed in lung cancer separately and also with other CRD, despite that there are meta-analyses addressing acupuncture in advanced cancer. Not like pain, the clinical efficacy of acupuncture in the treatment dyspnea has not been widely accepted by the medical community while the evidence is promising. Research on this area is needed given the uneven quality of studies and some controversies. There are also controversies about the effects of acupuncture on breathing physiology, as a factor that contributes to dyspnea in CRD such as COPD^[40]^ and asthma,^[41]^ which need to be clarified. The current study will also assess clinical outcomes of exercise tolerance and quality of life that can be impacted by dyspnea. Systematically evaluate the efficacy and safety of acupuncture in the treatment of CRD with dyspnea will provide scientific evidence for its clinical application.

## Author contributions

CX, YL and HW conceived and designed the study. CX and YL wrote the protocol. CYL and YFL will conduct literature search and data extraction. JJF proposed the protocol design and modified the manuscript. All authors have read and approved the final manuscript. CX and JJF are the study guarantors.

**Formal analysis:** Chen-Yi Li.

**Software:** Ye-Fang Liu.

**Validation:** Ye-Fang Liu, Hua Wei.

**Visualization:** Hua Wei.

**Writing – original draft:** Chan Xiong, Yu Li.

**Writing – review & editing:** Juan-Juan Fu.
